# Usefulness of Interventions Using a Smartphone Cognitive Behavior Therapy Application for Children With Mental Health Disorders: Prospective, Single-Arm, Uncontrolled Clinical Trial

**DOI:** 10.2196/60943

**Published:** 2025-07-29

**Authors:** Shinichiro Nagamitsu, Ayumi Okada, Ryoichi Sakuta, Ryuta Ishii, Kenshi Koyanagi, Chizu Habukawa, Takashi Katayama, Masaya Ito, Ayako Kanie, Ryoko Otani, Takeshi Inoue, Tasuku Kitajima, Naoki Matsubara, Chie Tanaka, Chikako Fujii, Yoshie Shigeyasu, Michiko Matsuoka, Tatsuyuki Kakuma, Masaru Horikoshi

**Affiliations:** 1 Department of Pediatrics, Faculty of Medicine, Fukuoka University, 7-45-1 Nanakuma, Jonan-ku, Fukuoka, 814-0180, Japan, 81 92-801-1011; 2 Department of Pediatrics, Okayama University Graduate School of Medicine, Dentistry and Pharmaceutical Sciences, Okayama, Japan; 3 Child Development and Psychosomatic Medicine Center, Dokkyo Medical University Saitama Medical Center, Shimotsugagun, Japan; 4 Department of Pediatrics & Child Health, Kurume University, School of Medicine, Kurume, Japan; 5 Nagasaki Prefectural Center of Medicine and Welfare for Children, Isahaya, Japan; 6 Department of Pediatric Allergy, Minami Wakayama Medical Center, Tanabe, Japan; 7 L2B Inc, Shibuya, Japan; 8 National Center for Cognitive Behavior Therapy and Research, National Center of Neurology and Psychiatry, Kodaira, Japan; 9 Department of Neuropsychiatry, Kurume University School of Medicine, Kurume, Japan; 10 Biostatistics Center, Kurume University, Kurume, Japan

**Keywords:** smartphone, cognitive behavioral therapy, application, adolescent, youth, teen, pediatric, mental health, psychoeducation, self-monitoring, questionnaire, depressive symptoms, effectiveness, Japan, statistical analysis, single-arm uncontrolled study, mobile phone

## Abstract

**Background:**

The prevalence of mental health disorders among children in Japan has increased rapidly, and these children often show depressive symptoms and reduced quality of life (QOL). We previously developed a smartphone-based self-monitoring app to deliver cognitive behavioral therapy (CBT), implemented it in healthy children, and reported its effectiveness for health promotion.

**Objective:**

This study aims to examine the usefulness of the CBT app for improvement in depressive symptoms and QOL in children with mental health disorders.

**Methods:**

The participants were 115 children with mental health disorders (eg, school refusal, orthostatic hypotension, eating disorders, developmental disorders, among others) and aged 12‐18 years. The CBT app–based program comprised 1 week of psychoeducation followed by 1 week of self-monitoring. After reading story-like scenarios, participants created a self-monitoring sheet with 5 panels: events, thoughts, feelings, body responses, and actions. All participants received regular mental health care from physicians in addition to the app-based program. To evaluate the participants’ depressive symptoms and QOL, Patient Health Questionnaire for Adolescents (PHQ-9A), Depression Self-Rating Scale for Children (DSRS-C), and Pediatric Quality of Life Inventory (PedsQL) were measured at the beginning of the intervention, and at 2 and 6 months thereafter. Questionnaire for Triage and Assessment with 30 items (QTA30), and Rosenberg Self-Esteem Scale (RSES) were also used to measure their health and self-esteem. Participants were divided into 4 groups on the basis of the PHQ-9A score (above or below the cutoff; PHQ-9A≥5 or PHQ-9A<5) and completion or noncompletion of the CBT app–based program (app [+] or app [-]). The primary outcome was improvement in the DSRS-C score, and secondary outcomes were improvement in other psychometric scales including PedsQL, QTA30, and RSE. A paired-samples *t* test was used for statistical analysis. The Medical Ethics Committee of Fukuoka University Faculty of Medicine (approval U22-05-002) approved the study design.

**Results:**

There were 48, 18, 18, and 7 participants in the PHQ-9A≥5 app (+), PHQ-9A≥5 app (-), PHQ-9A<5 app (+), and PHQ-9A<5 app (-) groups, respectively. A total of 24 participants dropped out. No improvement in the DSRS-C score was observed in all groups. However, PedsQL scores improved significantly at 2 and 6 months in the PHQ-9A<5 app (+) group (*t*_17_=6.62; *P*<.001 and *t*_17_=6.11; *P*<.001, respectively). There was a significant positive correlation between the PHQ-9A scores and the number of self-monitoring sheets completed.

**Conclusions:**

The CBT app was useful for improving PedsQL scores of children with mental health disorders. However, a higher-intensity CBT program is necessary for more severely depressed children.

## Introduction

The prevalence of mental health disorders among children has increased rapidly, and mental health care for children is an increasingly important part of pediatric health care delivery [1,2]. The number of children with school refusal is rapidly rising, and the “hikikomori” (severe social withdrawal) phenomenon may become more common in the postpandemic era [3]. In addition, it has been reported that COVID-19 has led to an increase in the number of children worldwide with depression, eating disorders, and sleep disorders [4-7]. Disability-adjusted life years of Japanese adolescents indicated that mental health disorders accounted for approximately 20% of the burden of disease [8]. Children with mental health disorders are more prone to experiencing depressive symptoms, which in turn can lead to a decline in their quality of life (QOL) [9-11] and potentially increase their risk of suicide. Suicide has become the leading cause of death among adolescents in many countries [12]. Therefore, it is important to establish a system for the treatment of these children’s mental health disorders.

Cognitive behavioral therapy (CBT) is one of the most effective means of treating mental health disorders in children. CBT, which focuses on improving the emotional state by changing thinking and behavior patterns, has been applied to a range of mental health disorders including depression, anxiety disorders, insomnia, and eating disorders [13-15]. It has also been reported that CBT improves QOL in patients with various physical conditions and mental health disorders [16]. In a study applying CBT in children with school refusal, improvements in school attendance, depression, and anxiety were reported [17]. However, the use of CBT treatments for mental health disorders in children and adolescents is often limited, where adolescents frequently refuse face-to-face psychotherapy because of embarrassment or time constraints. In recent years, mental health research has increased on CBT treatment provided via the internet and smartphones (ICBT) as an alternative to face-to-face CBT treatment [16,18-21], and the effectiveness of ICBT is reportedly no different from that of face-to-face CBT [22]. According to Aemissegger et al [23], the baseline characteristics of patients in internet-based and face-to-face intervention trials revealed no significant differences. However, internet-based trials showed a longer duration of depression and a lower proportion of patients with previous treatment experience, suggesting that such trials may attract patients who tend to delay seeking treatment [23].

ICBT includes therapist-guided ICBT and unguided ICBT, which does not involve a therapist. Guided ICBT provides individualized support and is considered highly effective, but the time of use is restricted. Unguided ICBT, on the other hand, offers convenience but no individualized guidance and is strongly dependent on the motivation of the user. In both forms of ICBT, while their effectiveness has been recognized, there are instances where the quality is lacking or randomized controlled trials (RCTs) meeting adequate standards have not been carried out [24-26]. However, time and effort tend to be spent on children’s mental illness in primary care [27], and it is hoped that ICBT will play a supplementary role to general practice.

We previously developed a smartphone-based self-monitoring application to deliver CBT (CBT app), implemented it in 217 healthy children, and reported its effectiveness for health promotion [28]. The CBT app was highly effective in terms of providing users with self-monitoring skills and reducing depressive symptoms. This study sought to examine the usefulness of the CBT app for improvement of depressive symptoms and QOL in children with mental health disorders including school refusal, eating disorders, and developmental disorders, among others. The primary outcome of this intervention is the improvement in the Depression Self-Rating Scale for Children (DSRS-C) scores after implementation of the CBT app.

## Methods

### Study Design

We conducted a prospective, single-arm clinical trial involving 115 children with mental health disorders aged 11‐18 years (mean age 14.9 SD 1.6 y). The trial was registered in the University Hospital Medical Information Network Clinical Trials Registry (registration UMIN000046775). Participants were enrolled from 6 collaborating institutions, and there were 49 and 66 male and female participants, respectively. The primary diagnoses were school refusal (n=54), orthostatic dysregulation (n=15), eating disorders (n=13), developmental disorders (n=10), irritable bowel syndrome (n=5), sleep disorders (n=5), somatoform disorders (n=4), obsessive-compulsive disorder (n=3), and other mental health disorders (n=6). The diagnoses were carried out entirely by pediatricians and conducted in accordance with the Japanese version of the Pediatric Psychosomatic Medicine Guidelines and the *Diagnostic and Statistical Manual of Mental Disorders, Fifth Edition*. Following the diagnosis, the primary physician introduced the study to the patient and their family, explained the use of the app as part of routine care, and obtained their consent. The app itself did not provide any information about the primary illness. Postdiagnosis interventions focused on disease education, lifestyle management guidance, dietary and sleep counseling, as well as directive counseling. Participants who were receiving medication did not change their medications during the study period. Participants were divided into 4 groups on the basis of the PHQ-9A score (above or below the cutoff; PHQ-9A≥5 or PHQ-9A<5) and completion or noncompletion of the CBT app-based program (app [+] or app [-]. PHQ-9A score of 5 or higher (PHQ-9A≥5) is considered indicative of a tendency toward depression. The program completion was verified on the basis of server records.

### Ethical Considerations

The design of this study and the procedures for obtaining informed consent were approved by the Medical Ethics Committee of Fukuoka University Faculty of Medicine (approval: U22-05-002). Informed consent was obtained from the children and their guardians, and the electronic consent forms were stored on a secure server. The research institution managed personal information by creating a correspondence table that linked participant names to research identification numbers, with the file protected by a password. Information provided by the collaborating research institutions to the primary research institution was anonymized, with participants identified only by their research identification numbers. As compensation, each participant received a US $15 QUO card per visit, provided directly to the individual. Payments were made three times: at the initial visit, 2 months later, and 6 months later.

### Procedure

The study inclusion criteria were as follows: (1) aged 11‐18 years, (2) first visit to a collaborating institution within 6 months, (3) approval for concurrent psychiatric treatment by the attending physician, and (4) access to a smartphone or Wi-Fi network. After the participants and parents signed the informed consent form, the CBT app was installed on their smartphones. While using the CBT app at home, participants visited the hospital at 1‐2-month intervals to receive psychological counseling for their primary illnesses from their physicians. The observation period was 6 months, and psychometric scales were assessed at the beginning of the intervention and 2 and 6 months thereafter.

### CBT App

**Figure 1.**
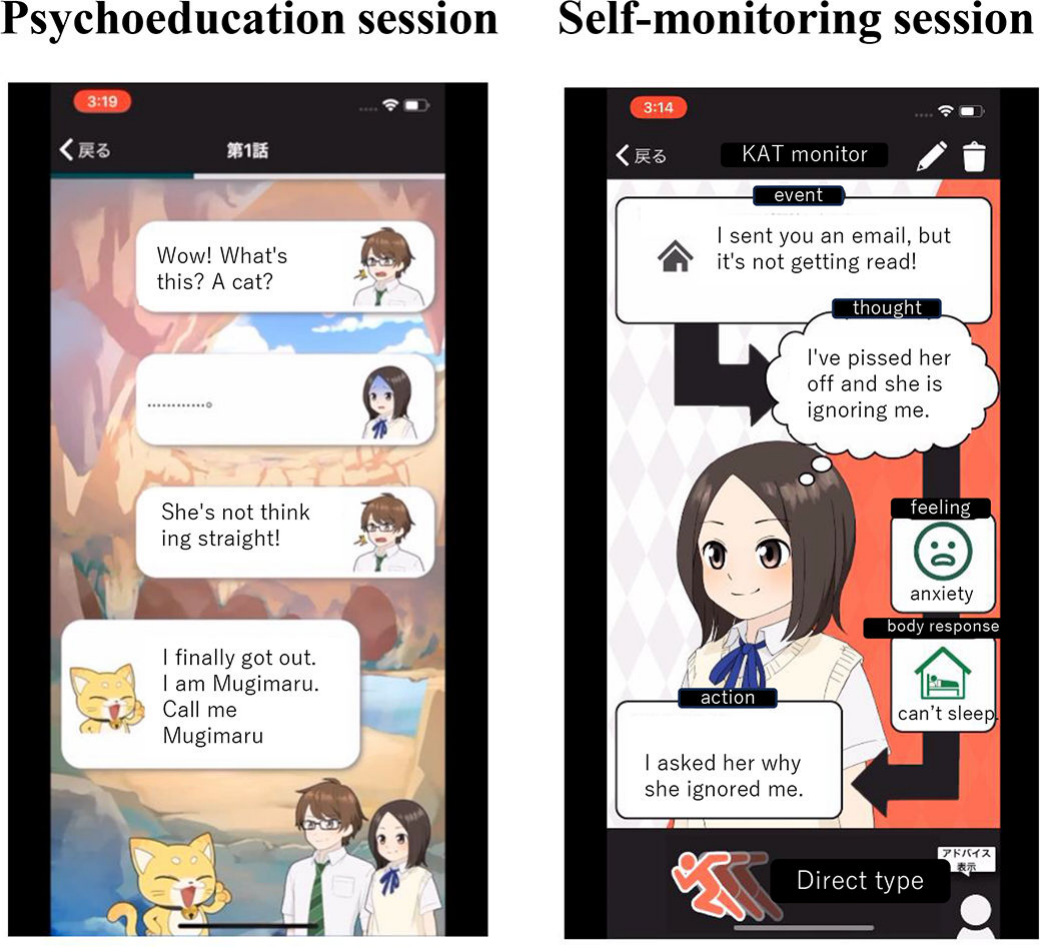
Screenshots of the smartphone cognitive behavior therapy app.

A smartphone-based CBT app, named Mugimaru (L2B Corporation). The program based on the app was described in detail previously [28]. Briefly, the program comprised 1 week of psychoeducation followed by 1 week of self-monitoring. Mugimaru presents story-like scenarios to provide psychoeducation, so that adolescents can easily understand the rationale of the CBT and are motivated to continue using the app. The story featured an adolescent boy, an adolescent girl, and a cat (Mugimaru). In the story, the boy and girl have troubles in their relationships with friends and concerns about their future. Mugimaru teaches them how feelings, thoughts, and actions mutually affect each other. Similarly, they learn that their feelings are associated with their thoughts and actions. The story consisted of 10 scenarios, and participants could browse 1‐2 scenarios each day. After reading a scenario, a new scenario could be read on the following day. The ending of the story was available 1 week after the participants had read the rest of the story. During the intervention period, participants completed several self-monitoring sheets comprising 5 panels: events, thoughts, feelings, body responses, and actions. The participants inputted their thoughts, feelings, body responses, and actions in association with daily events. In another window, the adolescents could input comments or advice for a friend who had experienced the same event. This input was used by adolescents to practice cognitive reappraisal and problem solving. Figure 1 shows screenshots of the smartphone CBT app. By repeatedly creating self-monitoring sheets, the adolescents could monitor their own experiences, develop solutions, and make necessary changes. The shortest time in which Mugimaru can be completed is 2 weeks. All of the data were stored in the main server, and the participants were informed in advance that only the principal investigator could view the data.

### Psychometric Scales

The following 4 psychometric scales were used for outcome assessment. DSRC-S and PedsQL were used for measurement of the extent of depressive symptoms and QOL, respectively. QTA30 and RSES were used for measurement of health scores and self-esteem, respectively.

#### Depression Self-Rating Scale for Children (DSRS-C)

The DSRS-C is an 18-item self-report questionnaire that measures depressive symptoms [29]. Participants are asked to select 1 of 3 response options for each item: “most of the time” (score=2), “sometimes” (score=1), or “never” (score=0). The maximum score is 36, and higher scores indicate stronger depressive tendencies. The Japanese version of the DSRS-C has good reliability and validity [30]. The cutoff score for possible depression on the Japanese version is 16 points.

#### Pediatric Quality of Life Inventory (PedsQL)

The PedsQL is a brief measure of adolescents’ health-related quality of life [31]. The 23 items are distributed among 4 generic core scales: physical functioning, emotional functioning, social functioning, and school functioning. Items are scored as 0 (“never”), 1 (“almost never”), 2 (“sometimes”), 3 (“often”), or 4 (“almost always”). The total scale score is calculated as the mean score of all the items transformed to a 0‐100 scale. Higher scores indicate better health-related quality of life. The Japanese version of the PedsQL has good reliability and validity [32].

#### QTA30

The QTA30, a psychosomatic evaluation scale for pediatric populations created by the Japanese Society of Psychosomatic Pediatrics [33], is a standardized tool for triage and assessment of pediatric patients with mental health disorders that takes Japanese cultural and social factors into account. The QTA30 comprises 4 scales: physical symptoms, depression symptoms, sense of self-efficacy, and anxiety symptoms. Higher scores on all scales correlate with poorer mental health. A total score of ≥37 points is considered to indicate poor mental health.

#### Rosenberg Self-Esteem Scale (RSES)

The RSES is the most widely recognized and used measure of global positive and negative attitudes toward the self [34]. It comprises 10 items, and responses are provided via a 4-point Likert scale (4, “strongly agree”; 3, “agree”; 2, “disagree”; and 1, “strongly disagree”). Negatively worded items are reverse scored, and total scores range from 10 to 40. Higher scores reflect greater levels of self-esteem. The Japanese version of the RSES has good reliability and validity [35].

### Data Analysis

As stated previously, the primary outcome of this study is the improvement in DSRS-C scores after completion of the CBT app-based program. The secondary outcomes were improvements in other psychometric scales including PedsQL, QTA30, and RSE. A paired-samples *t* test was used for statistical analysis. The mean baseline PHQ-9A scores between the app completion and noncompletion were compared. In addition, we explored the relationship between participants’ suicidal ideation and their completion or noncompletion of the app. To assess this, we calculated the number of individuals who selected “More than half the days” or “Nearly every day” for the PHQ-9A item, “Thoughts that you would be better off dead or of hurting yourself in some way,” and analyzed it in relation to their completion of the app. In addition, to determine the effect of app implementation for PHQ-9A score, multiple linear regression models were tested with the change in depression score (DSRS-C) or the change in QOL score (PedsQL) as the dependent variable and the PHQ-9A score and app implementation as independent variables. We also evaluated the correlations between the number of self-monitoring sheets completed by participants and their psychometric scale scores.

## Results

**Figure 2.**
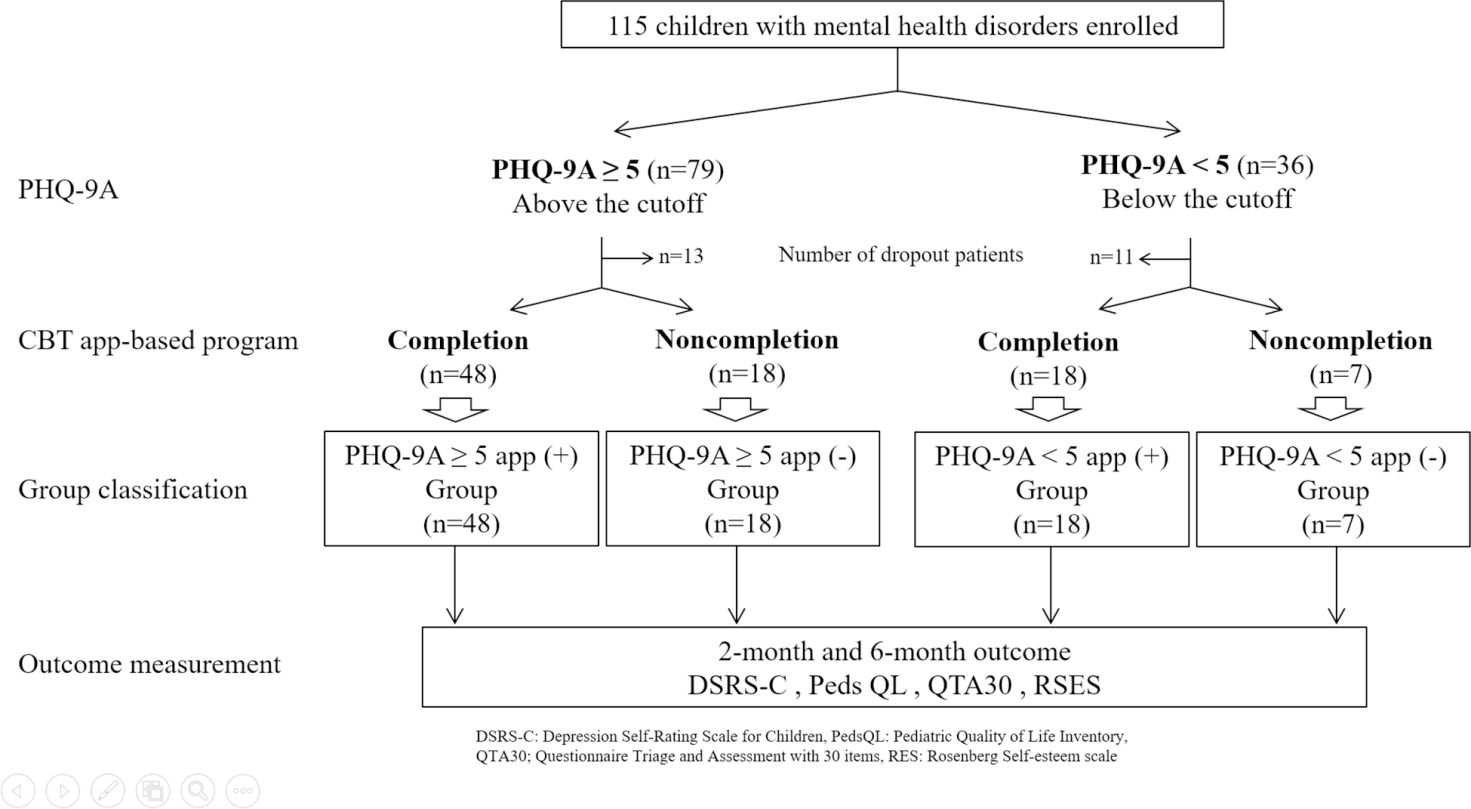
Participant flow chart. CBT: cognitive behavioral therapy; PHQ-9A: Patient Health Questionnaire for Adolescents.

In total, 115 children with mental health disorders participated in this study. According to the PHQ-9A data, 79 of the children showed PHQ-9A scores above the cutoff (PHQ-9A≥5), and 36 were PHQ-9A scores below the cutoff (PHQ-9A<5; see (Figure 2). Although 91 participants completed the 6-month outcome measurements, 24 dropped out (13 participants with PHQ-9A≥5 and 11 participants with PHQ-9A<5) before the 6-month measurements. Of the 91 participants who completed the 6-month outcome measurements, 66 also completed the CBT app-based program (48 participants with PHQ-9A≥5 and 18 participants with PHQ-9A<5); of the remaining 25 participants, 18 participants were PHQ-9A≥5 and 7 participants were PHQ-9A<5. The 91 participants who completed the 6-month outcome measurements were divided into 4 groups on the basis of PHQ-9A score (above or below the cutoff; PHQ-9A≥5 or PHQ-9A<5) and completion or noncompletion of the CBT app-based program (PHQ-9A≥5 app [+], PHQ-9A≥5 app [-], PHQ-9A<5 app [+], and PHQ-9A<5 app [-] groups). The primary diagnosis was not related to whether they completed the app. Table 1 shows the preintervention scores on each psychological scale.

**Table 1.** Comparison of the mean scores for each psychometric scale between baseline and follow-up measurements in 4 groups, categorized by the Patient Health Questionnaire for Adolescents (PHQ-9A) cutoff value and completion or noncompletion of the app program.

Follow-up period	PHQ-9^a^A≥5						PHQ-9A<5					
	App completion (n=48), mean (SD)	*t* test (*df*)	*P* value	App noncompletion (n=18), mean (SD)	*t* test (*df*)	*P* value	App completion (n=18), mean (SD)	*t* test (*df*)	*P* value	App noncompletion (n=7), mean (SD)	*t* test (*df*)	*P* value
DSRS-C^b^												
Baseline	18.77 (5.33)	—	—	17.83 (5.53)	—	—	10.17 (3.24)	—	—	11.57 (6.32)	—	—
2-month	18.75 (5.51)	0.03 (47)	.98	16.22 (5.81)	1.68 (17)	.11	8.83 (4.63)	1.90 (17)	.07	10.86 (3.29)	0.47 (6)	.65
6-month	19.04 (5.06)	0.39 (47)	.70	15.72 (5.68)	1.61 (17)	.13	8.56 (5.07)	1.87 (17)	.08	9.14 (4.45)	0.98 (6)	.37
PedsQL^c^												
Baseline	64.22 (17.53)	—	—	67.81 (13.01)	—	—	84.96 (8.42)	—	—	78.57 (12.13)	—	—
2-month	67.44 (17.03)	1.85 (47)	.07	71.26 (11.57)	1.59 (17)	.13	92.57 (7.44)	6.62 (17)	<.001	83.39 (11.72)	1.25 (6)	.26
6-month	71.22 (14.55)	3.36 (47)	.002	75.00 (10.67)	2.20 (17)	.04	94.75 (5.96)	6.11 (17)	<.001	81.21 (10.70)	0.54 (6)	.61
QTA30^d^												
Baseline	54.33 (14.19)	—	—	55.61 (12.35)	—	—	27.94 (12.13)	—	—	25.43 (6.02)	—	—
2-month	52.06 (18.01)	1.19 (47)	.24	49.78 (10.82)	2.03 (17)	.06	21.78 (12.76)	2.32 (17)	.03	26.43 (8.20)	0.34 (6)	.75
6-month	47.56 (16.12)	3.08 (47)	.003	47.11 (16.85)	2.44 (17)	.03	26.56 (22.43)	0.23 (17)	.82	23.43 (11.83)	0.42 (6)	.69
RSES^e^												
Baseline	20.85 (4.31)	—	—	21.50 (4.83)	—	—	27.94 (3.90)	—	—	26.57 (5.97)	—	—
2-month	20.40 (4.46)	0.96 (47)	.34	21.22 (4.18)	0.32 (17)	.76	28.11 (4.71)	0.25 (17)	.81	25.14 (3.76)	0.81 (6)	.45
6-month	20.38 (4.57)	0.76 (47)	.45	20.78 (3.59)	0.64 (17)	.53	27.94 (5.22)	0.00 (17)	≥.99	26.57 (3.87)	0.00 (6)	≥.99

a PHQ-9A; Patient Health Questionnaire-9.

b DSRS-C: Depression Self-Rating Scale for Children.

c PedsQL: Pediatric Quality of Life Inventory

d QTA30; Questionnaire Triage and Assessment with 30 items.

e RSE: Rosenberg Self-esteem scale,

### Outcomes at 2 and 6 Months

**Figure 3.**
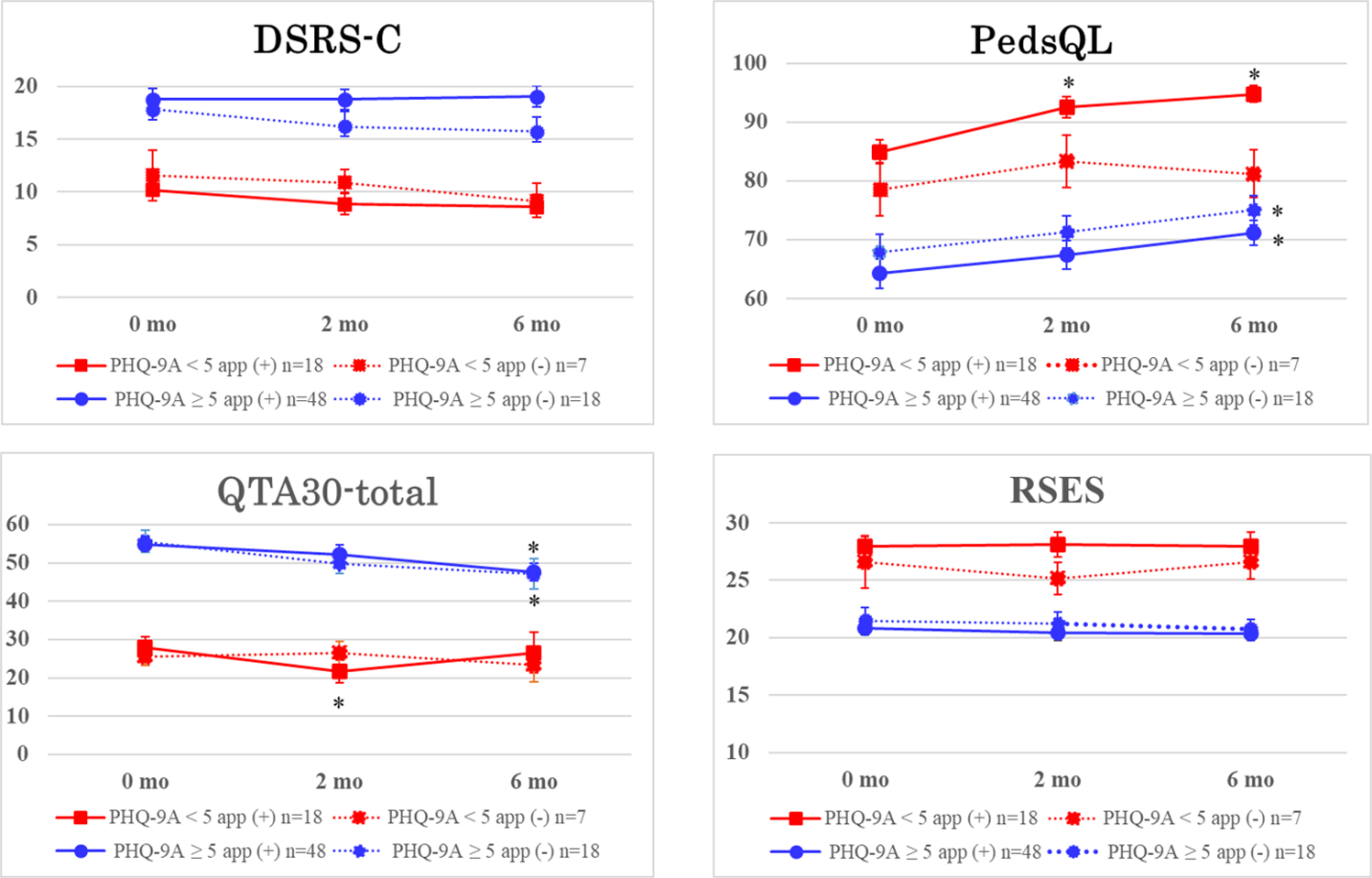
Changes in psychometric scores in the 4 groups during the follow-up period. DSRS-C: Depression Self-Rating Scale for Children; PHQ-9A: Patient Health Questionnaire for Adolescents; PedsQL: Pediatric Quality of Life Inventory; QTA30: Questionnaire for Triage and Assessment with 30 items; RSES: Rosenberg Self-Esteem Scale.

DSRS-C: Regardless of the completion or noncompletion of the CBT app-based program or whether the PHQ-9A value is above or below the cutoff, the DSRS-C scores did not improve significantly at 2 or 6 months (see Table 1 and Figure 3).

PedsQL: In the participants with PHQ-9A ≥5, the PedsQL scores at 6 months improved significantly regardless of the completion or noncompletion of the CBT app-based program. In the participants with PHQ-9A <5, the PedsQL scores at 2 and 6 months improved significantly only among those who completed the program.

QTA30: In the participants with PHQ-9A <5, the QTA30 score at 2 months improved significantly only among those who completed the program. In the participants with PHQ-9A ≥5, the QTA30 scores at 6 months improved significantly regardless of the completion or noncompletion of the CBT app-based program.

RSES: Regardless of the completion or noncompletion of the CBT app-based program or whether the PHQ-9A value is above or below the cutoff, the RSES scores did not improve significantly at 2 or 6 months.

### Comparison of the Mean Baseline PHQ-9A Scores Between the App Completion and Noncompletion Groups

In the group with PHQ-9A values above the cutoff (PHQ-9A≥5), the PHQ-9A score showed a tendency to be higher in the app noncompletion group (mean 13.33, SD 4.51), compared to the app completion group (mean 10.94, SD 5.02); however, the difference was not statistically significant (*t*_33,79_=1.86; *P*=.07). Conversely, in the group with PHQ-9A values below the cutoff (PHQ-9A<5), the PHQ-9A score (mean 3.29, SD 0.95) was significantly higher in the app noncompletion group (mean 1.72, SD 1.56) (*t*_18,17_=1.86; *P*<.01). For the PHQ-9A question, “Thoughts that you would be better off dead or of hurting yourself in some way,” 17 individuals responded with “More than half the days” or “Nearly every day.” Among them, 14 successfully completed the app.

### Multiple Linear Regression Analysis

Multiple linear regression models were used to test if PHQ-9A score and completion or noncompletion of the CBT app-based program significantly predicted the change in DSRS-C or the change in PedsQL score. These regression models were not statistically significant (see Tables2  3).

**Table 2.** Results of the regression model for changes in Depression Self-Rating Scale (DSRS) at 6 months of follow-up.

Terms	B^a^	SE	*t* test (*df*)	95% CI	*P* value
Intercept	1.210	1.659	0.729 (90)	−2.088 to 4.507	.47
PHQ-9A^b^	0.080	0.087	0.916 (90)	−0.094 to 0.253	.36
Implementation of the app	−2.125	1.173	1.182 (90)	−4.456 to 0.206	.07

a B: unstandardized coefficient.

b PHQ-9A: Patient Health Questionnaire for Adolescents.

**Table 3.** Results of the regression model for changes in Pediatric Quality of Life Inventory (PedQL) at 6 months of follow-up.

Terms	B^a^	SE	*t* test (*df*)	95% CI		*P* value
Intercept	7.887	4.365	1.807 (90)	–0.787 to 16.561		.07
PHQ-9A^b^	0.271	0.230	1.180 (90)	–0.185 to 0.727		.24
Implementation of the app	2.412	3.085	0.782 (90)	–0.8542 to 3.719		.44

a B:unstandardized coefficient.

b PHQ-9A: Patient Health Questionnaire for Adolescents.

### Correlation Between the Number of Monitoring Sheets Completed and Baseline Psychometric Scale Scores

**Figure 4.**
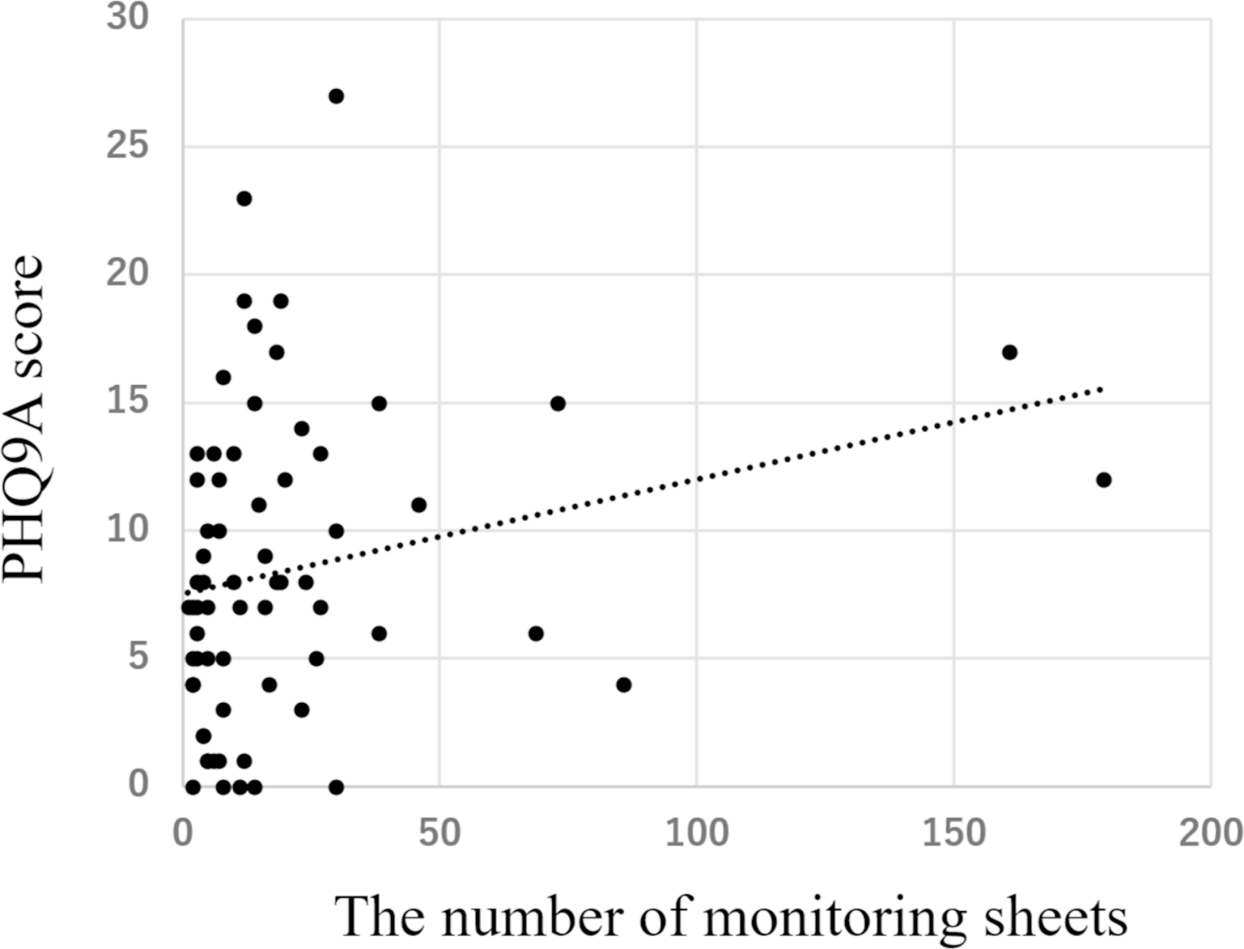
Correlation between the number of self-monitoring sheet completed by the participants and the PHQ-9A score. PHQ-9A: Patient Health Questionnaire for Adolescents.

There was a significant positive correlation between the PHQ-9A score at baseline and the number of monitoring sheets completed (see Figure 4).

## Discussion

This single-arm clinical trial demonstrated the usefulness of our CBT app-based program in terms of improving QOL (PedsQL) and health (QTA30) scores among children with mental health disorders who exhibited PHQ-9A scores below cutoff (PHQ-9A<5). However, children with PHQ-9A scores above the cutoff (PHQ-9A≥5) also showed improvements in QOL (PedsQL) and health (QTA30) scores at 6 months, even in the group that did not use the app. The app did not demonstrate effectiveness in improving depressive symptoms (DSRS-C) or self-esteem (RSE) at any time point. While a higher-intensity CBT program is necessary for more severely depressed children, this unguided app may play a supplementary role in the treatment of children with mental health disorders.

The improvement in depressive symptoms through the use of the app in children with mental health disorders was the primary outcome of this study; however, the app failed to show improvement in DSRS-C scores at any point, regardless of the initial PHQ-9A value. Although the CBT app has potential as an easily accessible, low-intensity intervention, the app might not be sufficient to reduce depressive symptoms in children with mental health disorders. Skar et al [36] analyzed the factors associated with nonresponse among children receiving CBT and identified higher baseline stress and older age as significant predictors; they also identified high intensity of the CBT intervention as key to prevent nonresponse. Nevertheless, the usefulness of app-based low-intensity CBT has also been noted. A meta-analysis of 66 randomized controlled trials of app-supported smartphone interventions for mental health problems found no significant difference in effectiveness versus active face-to-face interventions, showing the potential of apps to serve as cost-effective, easily accessible, low-intensity interventions for those who cannot receive standard high-intensity psychological treatment [37]. Adding standard CBT after low-intensity CBT was also reportedly effective in improving anxiety disorders in children [38,39]. The present CBT app consisted of 2 modules (psychoeducation and self-monitoring); additional modules including cognitive restructuring and behavioral activation are necessary to increase the effectiveness of the CBT app for children with more severe depression.

A notable finding of this study was improvements in PedsQL scores at 2- and 6-month follow-ups among children with PHQ-9A scores below the cutoff (PHQ-9A<5), achieved through the use of the app. The mechanism by which CBT improves QOL is believed to occur alongside improvements in depressive symptoms and self-esteem facilitated by CBT [40]. In this study, however, neither improvements in depressive symptoms nor self-esteem were observed, suggesting that the cartoon-style psychoeducational stories may have activated behavioral activation, a component of CBT, in the participants. On the other hand, improvements in PedsQL associated with the app were also observed at the 6-month follow-up among children with PHQ-9A scores above the cutoff (PHQ-9A≥5); however, similar improvements were noted in children who did not complete the CBT app-based program. As a medical examination was also performed and psychosocial counseling was provided in conjunction with the CBT app, it was not possible to evaluate the effects of the CBT app when analyzing the changes in the PedsQL scores in children with PHQ-9A ≥5. In general, smartphone and internet-based CBT apps are of low intensity, and their effects do not persist for a long time [41]; this likely explains why many intervention studies have 3-month outcome assessment periods [42]. In practice, it is difficult to provide treatment for mental health disorders using an app alone, that is, without also providing psychosocial counseling, so intervention studies comparing groups receiving psychosocial counseling with and without an app-based intervention are necessary to precisely determine the effects of apps.

One of the goals of this study was to learn about the differences in clinical characteristics between the CBT app-based program completion and noncompletion groups. In general, the noncompletion rate for smartphone- and web-based CBT is between 10% and 30% [43-45]. Predictors of discontinuation of CBT app use include worse physical health, lower education level, cognitive deficits, and less positive expectations of the outcome [43,46,47]. More severe pretreatment symptoms, comorbidities, lack of social support, and parental conflict are also associated with nonresponse to CBT [43,48-51]. In our study, 24 participants (20.9%) dropped out and another 25 (21.7%) discontinued the CBT app-based program. Among the latter cases, the PHQ-9A scores showed a trend toward being higher in those who did not complete the program compared with the scores among those who completed it. On the other hand, participants with strong suicidal ideation were more likely to complete the app. Furthermore, given that the participants with higher PHQ-9A scores completed more monitoring sheets, it appears that, although individuals with more severe depressive symptoms may be more likely to use a CBT app initially, they may also be more likely to give up. A stepped-care approach, which begins with lower-intensity internet-delivered CBT and then proceeds to more intensive treatments with face-to-face therapist involvement, could be effective for those who might otherwise show a poor response [38,39].

A few limitations of this study need to be addressed. First, this was a single-arm clinical trial involving children with mental health disorders who presented with varying PHQ-9A scores; a control group not using the app derived from the same population would be needed to precisely determine the effects of our CBT app. In the future, studies using randomized controlled trials (RCTs) with control groups are anticipated to provide more comprehensive and robust evidence. Second, the inclusion of participants with many disorders, such as school refusal, eating disorders, and developmental disorders, among others, may have led to variability in the results; conducting an intervention study including a population with a single disorder may have increased the reproducibility of the results. Third, 45 of the 115 participants dropped out of the study or discontinued their app use. There was a greater tendency toward dropout or discontinuation of the app among participants without depressive symptoms, and it is necessary to devise ways to encourage them to continue using the program.

Our CBT app-based program was useful for improving the QOL of children with mental health disorders with PHQ-9A<5. The program also seemed to be useful for children with PHQ-9A≥5, although the importance of regular psychosocial counseling is undeniable. A higher-intensity CBT program is necessary for more severely depressed children. By integrating this app into routine clinical practice, it serves as a supplementary tool to therapy, based on the principles of CBT, allowing individuals to observe their own mental states from a broader perspective.

## Abbreviations

CBT: cognitive behavioral therapy
DSRS-C: Depression Self-Rating Scale for Children
PedsQL: Pediatric Quality of Life Inventory
PHQ-9A: Patient Health Questionnaire for Adolescents
QOL: quality of life
QTA30: Questionnaire for Triage and Assessment with 30 items
RSES: Rosenberg Self-Esteem Scale
